# Activation of cell migration via morphological changes in focal adhesions depends on shear stress in MYCN-amplified neuroblastoma cells

**DOI:** 10.1098/rsif.2018.0934

**Published:** 2019-03-06

**Authors:** Takumi Hiraiwa, Takahiro G. Yamada, Norihisa Miki, Akira Funahashi, Noriko Hiroi

**Affiliations:** 1 Department of Biosciences and Informatics, Keio University, Kanagawa, Japan; 2 Department of Mechanical Engineering, Keio University, Kanagawa, Japan; 3 Department of Pharmacy, Sanyo-Onoda City University, Yamaguchi, Japan

**Keywords:** neuroblastoma, biomechanics, microfluidic device, metastasis, focal adhesion, shear stress

## Abstract

Neuroblastoma is the most common solid tumour of childhood, and it metastasizes to distant organs. However, the mechanism of metastasis, which generally depends on the cell motility of the neuroblastoma, remains unclear. In many solid tumours, it has been reported that shear stress promotes metastasis. Here, we investigated the relationship between shear stress and cell motility in the MYCN-amplified human neuroblastoma cell line IMR32, using a microfluidic device. We confirmed that most of the cells migrated downstream, and cell motility increased dramatically when the cells were exposed to a shear stress of 0.4 Pa, equivalent to that expected *in vivo*. We observed that the morphological features of focal adhesion were changed under a shear stress of 0.4 Pa. We also investigated the relationship between malignancy and the motility of IMR32 cells under shear stress. Decreasing the expression of MYCN in IMR32 cells via siRNA transfection inhibited cell motility by a shear stress of 0.4 Pa. These results suggest that MYCN-amplified neuroblastoma cells under high shear stress migrate to distant organs due to high cell motility, allowing cell migration to lymphatic vessels and venules.

## 1. Introduction

Migration of cancer cells towards lymphatic vessels and blood vessels is thought to be a critical step for distant metastasis [1,2]. In recent years, researchers have proposed that *in vivo* shear stress promotes the access of cancer cells to lymphatic vessels and blood vessels [3–7]. Cancer cells in the stromal space are exposed to shear stress caused by the interstitial flow [5]. Many types of cancer cell have the ability to respond to various scales of shear stress (table 1).

**Table 1.** Effect of shear stress on cancer cells.

cancer cell type	shear stress	response	reference
human MG63 and Saos2 osteosarcoma cells; SCC25 oral squamous carcinoma cells; SW1353 chondrosarcoma cells	1.2 Pa	fluid shear stress induces G_2_/M arrest	[8]
human PC3 prostate cancer cells	0.005 Pa	fluid shear stress promotes motility	[9]
human OVCAR-3 epithelial ovarian cancer cells	0.05–0.15 Pa	fluid shear stress promotes cell elongation and development of stress fibres	[10]
human MDA-MB-231 breast carcinoma cells	0.18 Pa	fluid shear stress alters cell morphology, polarization and focal adhesion formation	[11]
human COLO 205 colorectal adenocarcinoma cells; PC-3 prostate adenocarcinoma cells	0.04–0.2 Pa	fluid shear stress promotes TRAIL (apoptotic agent)-induced apoptosis	[12]

Neuroblastoma cells metastasize to distant organs [13], but the mechanism of this metastasis remains unclear. A previous study reported that shear stress promotes the adhesion of SK-N-SH neuroblastoma cells to endothelial cells [14], suggesting that neuroblastoma metastasis is induced by shear stress. Therefore, we hypothesized that the migration of malignant neuroblastoma cells is affected by shear stress.

The malignancy of neuroblastoma correlates with the amplification of MYCN, which encodes the transcription factor N-Myc [13,15]. In an orthotopic xenograft mouse model of human neuroblastoma, the MYCN-amplified neuroblastoma cell line IMR32 metastasizes to many distant organs, in contrast to single-copy MYCN neuroblastoma cells [16]. In *in vitro* experiments, MYCN amplification in neuroblastoma cells is related to cell motility and invasion ability [17,18].

Cell motility is regulated by focal adhesions (FAs) [19]. A FA is a complex structure at the basal surface of a cell, which is composed of various proteins such as *β*1 integrin and focal adhesion kinase (FAK) [20]. Morphologic features of FAs affect cell motility. The shape and axis orientation of FAs affect the direction and speed of cell migration, with an elongated shape and similar axis orientation tending to promote migration [21–23]. Furthermore, migration speed is positively associated with the length and area of FAs [21,22].

N-Myc regulates the expression of proteins in FAs. N-Myc inhibits the expression of *β*1 integrin [24] while promoting the expression of FAK [25]. Inhibition of *α*5*β*1 integrin using a blocking antibody has been shown to increase cell motility in single-copy MYCN neuroblastoma cells, but not in MYCN-amplified neuroblastoma cells [26]. On the other hand, FAK knockdown has been found to decrease cell motility in MYCN-amplified neuroblastoma cells, but not in single-copy MYCN neuroblastoma cells [27]. Cell adhesive force, which is reduced by the expression of FAK [28], is inversely correlated with cell motility in neuroblastoma cells [18,26]. These reports suggest that N-Myc regulates cell migration and cell adhesive force.

IMR32 is an MYCN-amplified neuroblastoma cell line. The expression of MYCN in IMR32 cells is 33 times higher than in single-copy MYCN neuroblastoma cell lines such as SK-N-SH [24]. N-Myc suppresses the expression of *β*1 integrin in IMR32 cells [24]. However, the relationship between MYCN expression and cell motility under shear stress in IMR32 cells is unclear.

We fabricated a logarithmic flow-rate device that can stimulate cells with logarithmically scaled shear stress [29] to investigate the relationship between shear stress and neuroblastoma cell motility. We found that high shear stress increases the motility of IMR32 cells and changes the morphological features of FAs, including the angle between the major axis of the FA and the direction of fluid flow, as well as the area, length and shape of the FA. Most IMR32 cells migrated downstream in the microchannel under high shear stress. Furthermore, we suggest that the high expression of N-Myc protein is related to the motility of IMR32 cells under high shear stress. These findings indicate that MYCN-amplified neuroblastoma cells efficiently reach lymphatic vessels and venules as a result of mechanical stimulation *in vivo*.

## 2. Material and methods

### 2.1. Cell culture

We obtained an IMR32 cell line (no. JCRB9050) derived from an abdominal neuroblastoma that was provided by the National Institutes of Biomedical Innovation, Health and Nutrition. Cell culture reagents for the cells were obtained from Wako Pure Chemical Industries (Osaka, Japan). The cells were routinely cultured in Dulbecco’s modified Eagle medium supplemented with 10% fetal bovine serum in a 5% CO_2_ incubator at 37°C. For immunofluorescence staining, cells were cultured in a glass-bottomed dish (IWAKI) coated with collagen (Cellmatrix Type IV, Nitta Gelatin).

### 2.2. SiRNA transfection

SiRNA oligonucleotides with two thymidine residues (dTdT) at the 3-end of the sequence were purchased from Nippon Gene. The sequence of the siRNA oligonucleotide targeting human MYCN was as follows: sense 5′-CGGAGATGCTGCTTGAGAA-3′ [30]. Transient transfections of 2.0 × 10^5^ cells were performed with siRNA (15 pmol) in Opti-MEM (Life Technologies) using Lipofectamine RNAiMAX (ThermoFisher Scientific). In this step, we used the reverse transfection method [31]. To study the specific effect of MYCN silencing, we prepared the following four groups (one experimental group and three different types of control): group 1 (MYCN-siRNA group; experimental), cells transfected with siRNA against human MYCN; group 2 (NC-siRNA group; control), cells transfected with a universal negative control siRNA that shows no homology to any eukaryote genes; group 3 (mock control group), cells treated with the transfection reagent and Opti-MEM without any siRNAs, to check the effect of the transfection reagent; group 4 (full control), cells received no treatment. After 2 days of siRNA treatment, cells were used for immunostaining and microfluidic culture.

### 2.3. Mask design

Our device consists of three layers: the cell culturing layer, the fluidic layer and the pneumatic layer. The cell culturing layer consists of fluidic regulation channels and cell-culture channels. The fluidic layer consists of two levels of channels, and the pneumatic layer consists of an air valve [32]. The film masks were designed using Inkscape (v. 0.48, http://www.inkscape.org) and purchased from Vanfu (Tokyo, Japan).

### 2.4. Device fabrication

SU-8 3010 (Newton, MA, USA) was applied to a glass wafer (S9111, Matsunami Glass, Osaka, Japan), which was then spun and baked at 100°C. The wafer was exposed to UV through the high-resistance channel mask using a desktop aligner (EMA-400, Union Optical, Tokyo, Japan), then baked at 60°C for 1 min and 100°C for 5 min. After baking, SU-8 3010 was again applied to the wafer, and it was again spun and baked at 100°C. The moderate-resistance channel mask was then aligned on the wafer, and the wafer was exposed to UV and baked at 60°C for 1 min and 100°C for 5 min. SU-8 3010 was once again applied to the wafer, and it was again spun and baked at 100°C. Finally, the low-resistance channel was aligned on the wafer, which was exposed to UV and baked at 60°C for 1 min and 100°C for 5 min. The wafer was then soaked in SU-8 developer. The parameters of the process described above are listed in table 2, as are the parameters for fabricating the fluidic and pneumatic layer moulds.

**Table 2.** Parameters of mould fabrication.

mask	resist	rotation speed (rpm)	softbake (min)	exposure dose (mJ cm^−2^)	postbake (60°C/100°C)
high-resistance channel	SU8 3010	3000	5	420	1 min/5 min
moderate-resistance channel	SU8 3010	2500	5	420	1 min/5 min
low-resistance channel	SU8 3010	2500	5	420	1 min/5 min
lower channel	SU8 3050	1300	20	420	1 min/8 min
upper channel	SU8 3050	1300	20	480	1 min/10 min
air valve^a^	SU8 3050	1300	20	480	1 min/10 min

^a^To produce 200 *µ*m height of mould, spin coating and softbake processes were performed twice.

### 2.5. Particle image velocimetry

We prepared 0.05% (v/v) fluorescent polystyrene beads (diameter 500 nm, Life Technologies, USA) in distilled water containing 0.1% Triton-X [33]. This solution was injected into the fluidic inlet at a flow rate of 5.0 or 10.0 *µ*l min^−1^ using a micro-syringe pump (Nexus 3000, Chemyx, USA). We used the ‘iterative PIV’ plugin [34], installed in Fiji [35], to measure the velocity of the fluorescent beads.

### 2.6. Cell culturing in the device

The device was sterilized with a 70% ethanol wash and in an autoclave (20 min at 120°C). The sterilized device was connected to a vacuum pump (DAP-15, ULVAC KIKO, Miyazaki, Japan) and placed on a collagen-coated glass substrate. To completely flush out air in the microchannel, the cell outlets were closed with plugs and the culture medium was perfused at a flow rate of 5.0 *µ*l min^−1^ for 1 h with a micro-syringe pump. After the device had been filled with the medium, the cell outlet plugs were removed and the cell suspension (2 × 10^6^ cells ml^−1^) was loaded into the fluidic inlet. After the cells had been loaded, the outlets were closed with the plugs. The device was placed in a 5% CO_2_ stage-top incubator (INUG2-ONICS, TOKAI HIT, Shizuoka, Japan) at 37°C. The cells were cultured at a flow rate of 0.0, 5.0 and 10.0 *µ*l min^−1^.

### 2.7. Immunofluorescent staining

Cells were fixed for 15 min in 4% paraformaldehyde (Wako). They were then permeabilized and blocked with 0.1% Tween-20 in phosphate-buffered saline (PBS) for 5 min and 3% bovine serum albumin (012-23881, Wako) in PBS for 30 min. The cells were incubated with mouse monoclonal anti-*β*1 integrin and rabbit polyclonal anti-FAK pY925 (1/250, Abcam, ab30394 and ab39967) for 1 h at room temperature. They were then washed twice with PBS and incubated with anti-mouse-IgG (H + L) secondary antibody conjugated with Alexa Fluor 568 and anti-rabbit-IgG (H + L) secondary antibody conjugated with Alexa Fluor 488 for 1 h at room temperature in the dark. Nuclei were visualized with Hoechst 33342 (Lonza).

For N-Myc protein staining, the fixed cells were permeabilized with 1% Triton-X for 1 h [30]. The cells were blocked with 3% bovine serum albumin for 30 min, and then incubated with mouse monoclonal anti-N-Myc (Santa Cruz, 1/100, sc-515099) for 1 h at room temperature. They were then incubated with anti-mouse-IgG (H + L) secondary antibody conjugated with Alexa Fluor 568 for 1 h at room temperature in the dark.

### 2.8. Image acquisition and processing

Images were acquired using an Olympus IX81 with Orca Flash 4.0 (Hamamatsu), or an Olympus IX71 with Orca R2 (Hamamatsu). For live-cell imaging of cell migration, 50 phase-contrast images were sequentially acquired at intervals of 5 min using a 10 × objective. The immunostained FAs were captured using a 100 × objective. The immunostained N-Myc protein was captured using a 40 × objective.

For analysis of the morphological features of FAs, we used the images of immunofluorescent-stained *β*1 integrin. The Gaussian blur (radius = 2 pixels) and the rolling ball algorithm (radius = 5 pixels) [36] were applied to reduce noise, before the images were binarized using Otsu thresholding [37]. Erode operation and watershed [38] were applied to the binarized images to separate unexpectedly combined objects. We measured the angle of the major axis, area, length, breadth and shape factor of the FAs using the ‘analyse particle’ plugin installed in Fiji. We excluded objects smaller than 1 [pixel × pixel]. The angle variance (*V*) was calculated using the following equation [39]:

2.1 V=1−M,

where *M* is the mean resultant length, which is defined by the following equation:

2.2 $$(M\cos\phi ,M\sin\phi )=\frac{1}{N_{\theta }}\left(\sum_{k}\cos(2\theta_{k}),\sum_{k}\sin(2\theta_{k})\right),$$

where *N*_*θ*_ is the sample size of the angle data. The angle data *θ*_*k*_(− 90° ≤ *θ*_*k*_ ≤ 90°) are a dataset of the angles of the *β*1 integrin clusters. We defined the angle of the flow direction as 0°. The shape factor is calculated using the following equation:

2.3 $$\text{Shape factor}=\frac{4\pi A}{P^{2}},$$

where *A* and *P* are the area and perimeter of FAs, respectively. FAs whose shape factor was 1.0 were excluded from the dataset, because a shape factor of 1.0 means a circle, whose angle cannot be measured.

For quantification of MYCN expression, we used the images of immunofluorescent-stained N-Myc. We manually set a region of interest (ROI) that enclosed IMR32 cells in the phase-contrast images. The N-Myc signal intensity was calculated by subtracting the background intensity from the mean intensity of the ROI.

To obtain the trajectory of cells, we tracked them manually using Fiji’s ‘Manual Tracking’ plugin.

### 2.9. Statistical analysis

To confirm that the data were normally distributed, we performed a Kolmogorov–Smirnov test, with a *p*-value < 0.05 considered significant. For multiple comparisons, a Welch’s *t*-test was performed, with Bonferroni’s correction applied. A statistically significant change was confirmed if the family-wise error rate was less than 0.05. To compare all other conditions to controls, we performed the Dunnett’s test, and *p*-values < 0.05 were considered significant [40]. All experiments were performed at least three times.

## 3. Results

### 3.1. Development and evaluation of the logarithmic flow-rate device

**Figure 1.**
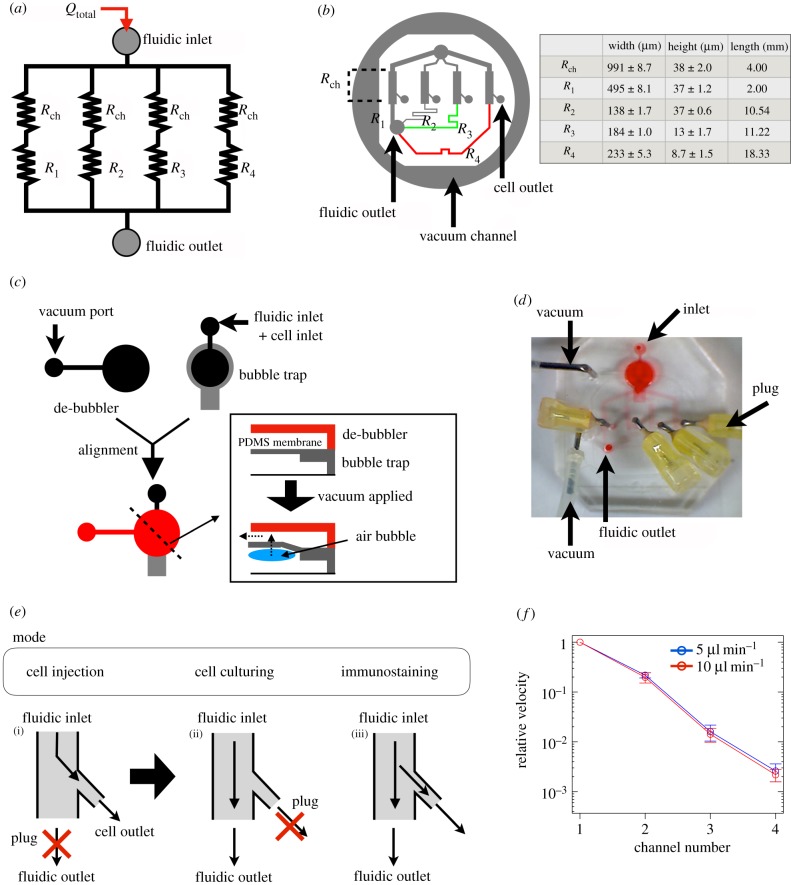
Design and evaluation of the logarithmic flow-rate device. (*a*) Schematic diagram of the logarithmic flow-rate device, showing four culture channels (*R*_ch_) and four logarithmically scaled resistors (*R*_1_–*R*_4_). (*b*) Design of the logarithmic flow-rate device. Colours indicate the height of the channel. The table on the right gives the dimensions of each channel. (*c*) Design of the bubble trap and the de-bubbler. The lower right panel shows a cross section of the device at the dashed line. Small bubbles are trapped at the PDMS membrane, which is deformed by applying a vacuum. The bubbles can then be removed by passing through the PDMS membrane. The dark coloured (black or red) and light grey sections are the de-bubbler and the bubble trap, respectively. (*d*) Photograph of the logarithmic flow-rate device. (*e*) Diagram of the operation modes of the device. The fluidic outlet was plugged when the cells were loaded (i). The cell outlet was plugged when the cells were cultured (ii). The fluidic outlet and the cell outlet were not plugged when the immunostaining was performed (iii). (*f*) Relative velocity for each culture-channel cell-culturing mode. The velocity was measured by particle image velocimetry. The upper (blue) and lower (red) lines indicate the relative velocities at flow rates of 5.0 *µ*l min^−1^ and 10.0 *µ*l min^−1^, respectively. Each data point shows the mean of the relative velocity. Error bars show the standard deviation. The experiment was performed three times. (Online version in colour.)

First, we fabricated a device for stimulating cells with logarithmically scaled shear stress [29]. The device consists of four cell-culture channels and four logarithmically scaled resistors (figure 1*a*). The flow rate in each cell-culture channel depends on the width (*w*), height (*h*) and length of the channels [29]. The dimensions of each channel were designed as shown in figure 1*b*. The device has a vacuum channel which can reversibly bond polydimethylsiloxane (PDMS) to a glass surface [32]. A bubble trap and a pneumatic de-bubbler were built into the device to remove small bubbles invading via the fluidic inlet (figure 1*c*) [41]. We set up an experimental system (figure 1*d*) and measured fluid velocities in the cell-culture channel during the cell culturing mode (figure 1*e*). It was confirmed that the relative velocities were logarithmically different and that the ratio of the relative velocities was constant at flow rates of 5.0 μl min^−1^ and 10.0 μl min^−1^ (figure 1*f*).

### 3.2. IMR32 cells exposed to high shear stress migrated downstream

We investigated whether the shear stress affected the motility of the IMR32 cells. Total flow rates were set at 0.0 *µ*l min^−1^ (static culture), 5.0 *µ*l min^−1^ and 10.0 *µ*l min^−1^. The flow rate in each cell culture channel (*Q*_*i*_) was calculated using the relative velocities (figure 1*f*), and then the shear stress on the wall (*τ*) was calculated according to the following equation [42]:

3.1 $$\tau =\frac{6\mu Q_{i}}{h^{2}w},$$

where *μ* is the fluid viscosity of the cell culture medium (0.0007 Pa s [43]). Values in figure 1*b* were used as the parameters (width and height) in equation (3.1).

**Figure 2.**
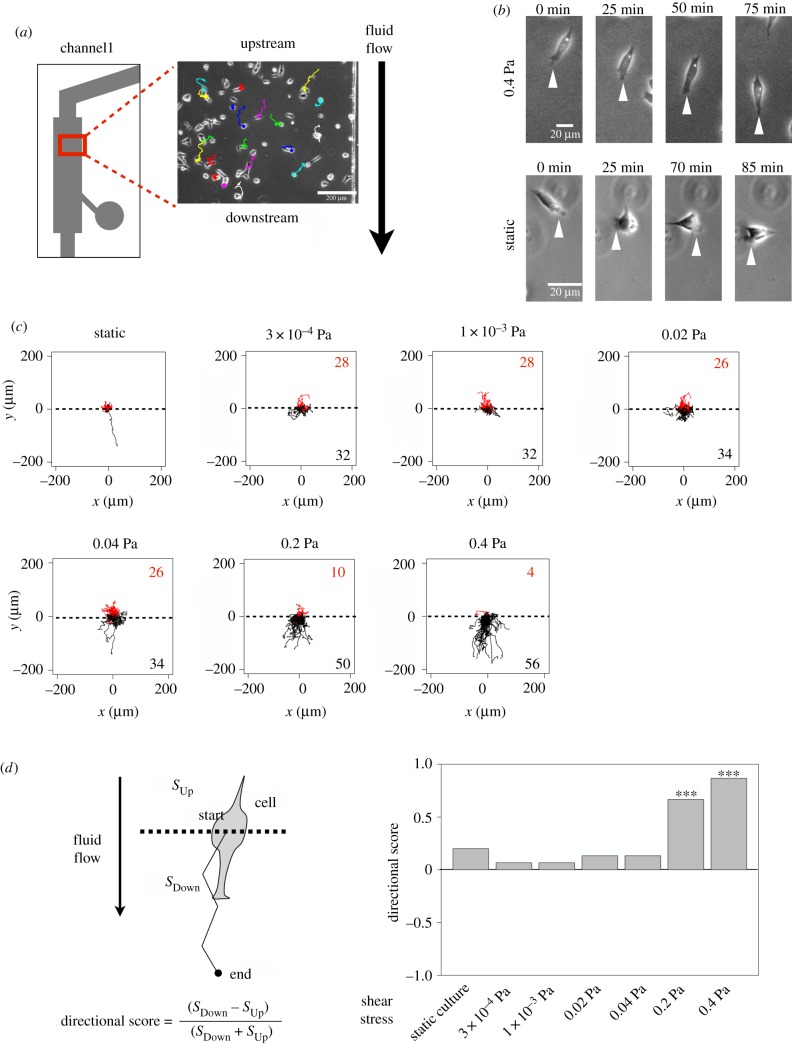
Cell trajectories in the logarithmic flow-rate device. (*a*) Phase contrast image of the perfusion culture in the cell-culture channel. Each colour represents a tracked cell trajectory. Scale bar =200 *µ*m. (*b*) Sequential images of cell migration under high shear stress or static conditions. Upper images show cell migration under a shear stress of 0.4 Pa. The top and bottom of the image are the upstream and downstream ends of the cell-culture channel, respectively. Lower images show cell migration under static culture. White arrowheads indicate the protrusion of the leading edge. (*c*) Cell trajectories under a range of shear stress conditions. Top and bottom sections of the plots are the upstream and downstream ends of the cell-culture channel, respectively. Red lines (above the dashed line at 0) depict trajectories whose ending position was upstream of the starting position. Black lines (below the dashed line at 0) depict trajectories whose ending position was downstream of the starting position. The numbers in each panel show the number of cells that migrated upstream (upper right, red) or downstream (lower right, black). In each panel, 60 cells were monitored for 245 min at intervals of 5 min. (*d*) Quantification of the direction of cell migration under each shear stress condition. Directional score is calculated by the equation shown at the bottom. *S*_Up_ and *S*_Down_ represent the number of cells that migrated upstream and downstream, respectively. To obtain the directional score, *S*_Up_ was subtracted from *S*_Down_ and divided by the total number of the cells. The right bar plot shows the directional score under each shear stress condition. A two-sided, exact binomial test was performed. ***p < 0.001. (Online version in colour.)

It has been reported that the direction of cell migration is regulated by the extension of the leading edge [44]. We monitored the cell migration for 245 min at 5 min intervals (figure 2*a*). Cells were manually tracked using Fiji. Under a shear stress of 0.4 Pa, the IMR32 cells formed their leading edge consistently on their downstream side (figure 2*b*, upper images). By contrast, under static culture, they formed their leading edges in random directions (figure 2*b*, lower images). Next, we analysed 60 trajectories and found that most of the cells migrated downstream under a shear stress of 0.4 Pa (figure 2*c*). The number of cells that migrated downstream was counted, and the tendency of the cells to migrate downstream was tested for statistical significance using a two-sided exact binomial test. This indicated that under a shear stress of 0.2 Pa or more, cells underwent directional migration (*p* < 0.001, figure 2*d*). By contrast, at a shear stress of below 0.04 Pa, cells migrated randomly (figure 2*d*). Combined, these results suggest that shear stress regulates the protrusion of the leading edge in IMR32 cells.

### 3.3. Descriptors of cell motility under shear stress

**Figure 3.**
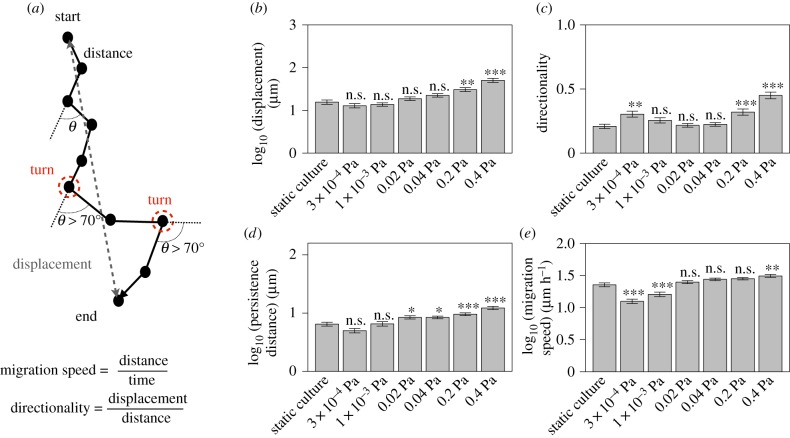
Descriptors of cell motility under each shear stress condition. (*a*) Schematic of analysis of cell trajectories. Displacement was the distance from the initial position to the final position of the measurement. Directionality was calculated by dividing the displacement by the distance. Persistence distance was the displacement between each turn, with a turn defined as a significant change in direction (greater than 70°). If there were no turns in the trajectory, the displacement between the initial and final positions was equal to the persistence distance. (*b*) Displacement under each shear stress condition. (*c*) Directionality under each shear stress condition. (*d*) Persistence distance under each shear stress condition. (*e*) Migration speed under each shear stress condition. All shear stress conditions were compared with the control group (static culture) using a Dunnett’s test. *p < 0.05, **p < 0.01, ***p < 0.001, n.s. not significant. Bars and error bars show the mean and the standard error. Sixty trajectories were used for this analysis. (Online version in colour.)

Next, we investigated the relationships between descriptors of cell motility and shear stress. The descriptors we used were displacement, directionality, persistence distance and migration speed [21], which were calculated as shown in figure 3*a*.

The average value of each descriptor under a shear stress of 0.4 Pa increased 1.37- to 3.23-fold relative to the static condition (displacement: 3.23 times greater; directionality: 2.15 times higher; persistence distance: 1.89 times greater; migration speed: 1.37 times higher; figure 3*b*–*d*). In particular, all descriptors increased significantly under a shear stress of 0.4 Pa (Dunnett’s test; adjusted *p* < 0.05). This suggests that shear stress increases directionality, persistence distance and migration speed; as a result, the displacement of migrating cells is also increased.

### 3.4. Morphological changes in focal adhesions under shear stress

**Figure 4.**
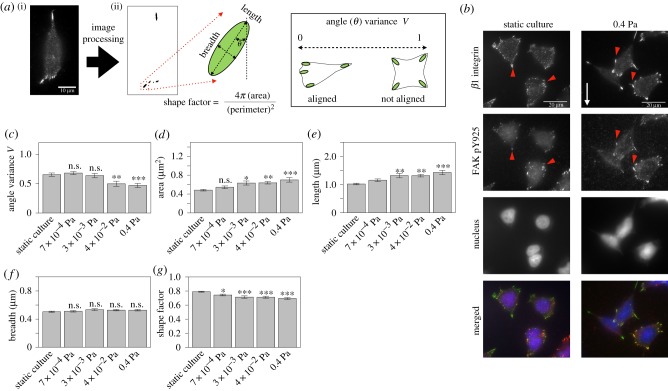
The effect of shear stress on FAs. (*a*) Schematic of the FA analysis (i). The angle, length, breadth and shape factor were measured by fitting an ellipse to the *β*1 integrin cluster in the image. Diagram of angle variance (*V*) (ii). A small angle variance indicates that the major axes of the FAs in a cell are aligned in the same direction. A large angle variance indicates that the major axes are oriented in random directions. (*b*) Immunofluorescent images of *β*1 integrin and FAK pY925. Right-hand images show the immunostained cells under a shear stress of 0.4 Pa, and left-hand images show the immunostained cells in the static culture treatment. The white arrow indicates the direction of culture medium flow. The arrowheads indicate the co-localization of *β*1 integrin and FAK pY925. (*c*–*g*) The morphological features under each shear stress condition. All shear stress conditions were compared with the static culture as the control group (*n* = 35, 37, 29, 32 and 39 cells for the static culture, 7 × 10^−4^ Pa, 3 × 10^−3^ Pa, 4 × 10^−2^ Pa and 0.4 Pa, respectively), using a Dunnett’s test. Bars and error bars show the mean and the standard error. *p < 0.05, **p < 0.01, ***p < 0.001. n.s. not significant. (Online version in colour.)

Cell migration speed, directionality and persistence distance are affected by the angle variance of the major axis of the FA, as well as the size and shape of the FA [21–23,45]. We therefore measured various morphological features of the FAs under shear stress: angle variance (*V*), area, length, breadth and shape factor of the *β*1 integrin cluster (figure 4*a*). The angle variance is the degree of variation in the angle (*θ*) of the major axis of the FAs (figure 4*a*(ii)). In this analysis, we defined *β*1 integrin clusters as the FAs because they were co-localized with FAK pY925 (figure 4*b*), which is a known component of FAs and promotes FA turnover, protrusion and cell migration [45].

We found that the FA angle variance and shape factor were decreased by increasing shear stress (figure 4*c*,*g*), and that FA area and length were increased (figure 4*d*,*e*). However, there was no significant difference in FA breadth under different shear conditions (Dunnett’s test; adjusted *p* > 0.05; figure 4*f*).

It has been reported that FAs with a small angle variance and decreased shape factor promote directional cell migration [21,23]. Furthermore, it has been reported that increases in the area and length of FAs promote cell migration speed [21,22]. In our study, the direction of cell migration was the same as the direction of fluid flow (figure 2). Combined, these results suggest that the mechanisms underlying the morphological changes in FAs contribute to changes in cell motility.

### 3.5. MYCN-siRNA transfection inhibits the alignment of the major axis of focal adhesions under the shear stress

**Figure 5.**
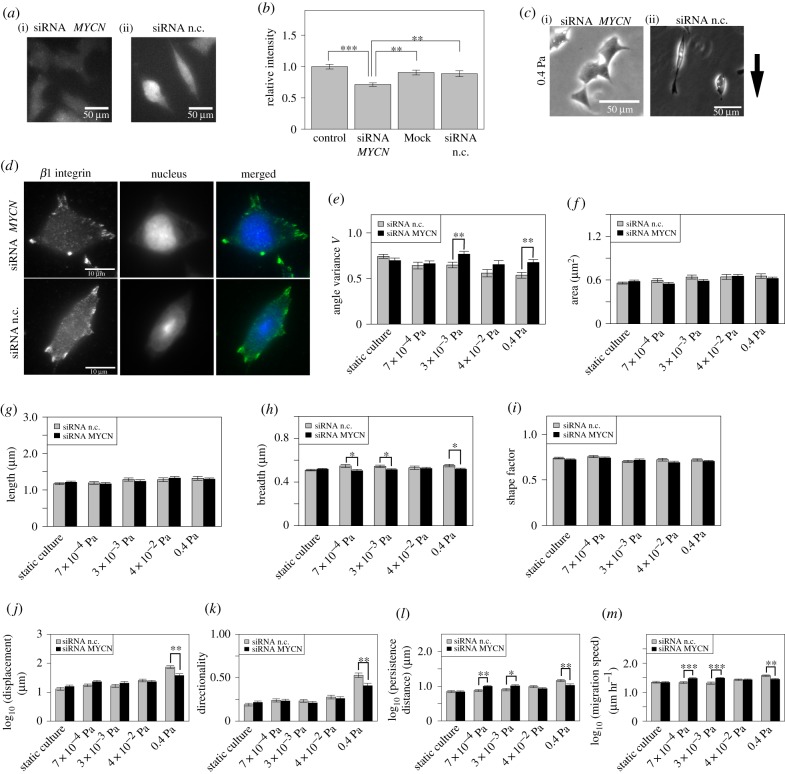
MYCN-siRNA transfection inhibits morphological changes in FAs under shear stress. (*a*) N-Myc protein was immunostained with an anti-MYCN antibody. The expression of N-Myc protein in the MYCN-siRNA transfection (i), and in the condition of the negative control siRNA (n.c., ii) treatments. (*b*) Quantification of the N-Myc protein expression. ‘Control’: no treatment; ‘Mock’: transfection-reagent treatment only. Welch’s *t*-test and Bonferroni’s correction were used. Experiments were replicated three times each. (*c*) Phase contrast image of IMR32 cells under high shear stress (0.4 Pa) after siRNA transfection. The black arrow indicates the direction of culture medium flow. (*d*) Fluorescence image of *β*1 integrin immunostained with an anti-*β*1 integrin antibody (upper images: MYCN-siRNA, lower images: negative control siRNA; both conditions under a shear stress of 0.4 Pa). The top and bottom sections of each image are upstream and downstream ends of the cell-culture channel, respectively. (*e*–*i*) Morphological features under each shear stress condition, for the MYCN-siRNA and negative control siRNA treatments. Results compared using Welch’s *t*-test (MYCN-siRNA: *n* = 37, 24, 22, 23 and 33 cells; negative control siRNA: *n* = 36, 31, 27, 27 and 36 cells). The displacement (*j*), directionality (*k*), persistence distance (*l*) and migration speed (*m*) were reduced under high shear stress (0.4 Pa) by MYCN-siRNA transfection. Bars and error bars show the mean and standard error. *p < 0.05, **p < 0.01, ***p < 0.001, n.s. not significant. (Online version in colour.)

We treated IMR32 cells with the MYCN-siRNA to investigate the relationship between the expression of N-Myc protein and cell migration under shear stress. Two days after the siRNA transfection, immunostaining of N-Myc protein in the static culture was performed, and it was confirmed that the expression of MYCN was reduced by 20–30% by the MYCN-siRNA transfection (figure 5*a*,*b*). Cells transfected with the MYCN-siRNA exhibited a nonsalaried cell shape under high shear stress (figure 5*c*(i)), whereas cells transfected with the negative control siRNA exhibited a polarized cell shape under these conditions (figure 5*c*(ii)). After the perfusion culture, immunostaining of *β*1 integrin was performed. The results indicated that the FAs were isotropically distributed in cells transfected with the MYCN-siRNA and subjected to high shear stress (figure 5*d*, upper images). By contrast, the major axes of the FAs in cells transfected with the negative control siRNA were aligned under the same culture conditions (figure 5*d*, lower images). We therefore hypothesized that the MYCN-siRNA treatment ameliorates some of the effects of shear stress on the morphological features of the FAs.

Next, we investigated the effects of MYCN-siRNA treatment on the morphological features of FAs under shear stress. We found that alignment of the FAs under shear stress was inhibited by MYCN-siRNA transfection (figure 5*e*), but that the area, length and shape factor were not significantly affected by shear stress (figure 5*f*,*gi*; adjusted *p* > 0.05). Interestingly, MYCN knockdown increased the breadth of FAs under shear stress (figure 5*h*).

Finally, we investigated the descriptors of cell motility under shear stress when the MYCN-siRNA or the negative control siRNA was added. We found that all the descriptors were significantly smaller under a shear stress of 0.4 Pa when MYCN-siRNA was added (figure 5*j*–*m*). The average value of each descriptor under a shear stress of 0.4 Pa increased 0.5110- to 0.772-fold relative to the negative control siRNA transfection (displacement: 0.511 times greater; directionality: 0.772 times higher; persistence distance: 0.727 times greater; migration speed: 0.747 times faster). The knockdown experiment therefore suggests that expression of N-Myc protein influences the morphological features of FAs under high shear stress, and that these morphological features affect cell motility.

## 4. Discussion

We found that the motility of IMR32 cells is affected by shear stress. In particular, high stress (0.4 Pa) changes all the cell motility descriptors (figure 3*b*–*e*). A shear stress of 0.4 Pa is comparable to shear stress values predicted in blood flow and interstitial flow in tumours [5,6,46]. Tumour cells have the ability to survive even when exposed to very high shear stress (≈600 Pa) [47]. These findings suggest that shear stress increases the motility of MYCN-amplified neuroblastoma cells *in vivo* without including cell death.

We also found that the directionality and persistence distance were changed under the low shear stress (figure 3*c*,e). This is assumed to be the effect of high hydrostatic pressure. The hydrostatic pressure in a culture channel is higher with a low flow rate than with a high one (Bernoulli’s principle). The motility of vascular smooth muscle cells is affected by hydrostatic pressure [48]. Thus, we conclude that the higher hydrostatic pressure affected the directionality and persistence distance of IMR32 cells.

Under the high shear stress conditions (0.4 Pa), the cells migrated downstream of the medium flow (figure 2*d*). We confirmed that *β*1 integrin localized on the downstream side significantly under this condition (electronic supplementary material, figure S1). In the IMR32 cells, the *β*1 integrin clusters were co-localized with FAK pY925 clusters (figure 4*b*). FAK pY925 promotes protrusion during cell migration [45]. Our analysis therefore suggests that the downstream migration of IMR32 cells under high shear stress is promoted by the localization of FAK.

We found that the directionality and migration speed increased (figure 3*c*,e), and that the angle variance of the FA axes was smaller under high shear stress (figure 4*c*). It is known that shear stress activates integrins and causes the alignment of actin filaments in the direction of fluid flow [49]. The major axes of FAs align along actin filaments [50]. Previous research found that the directionality increased when FAs in MDA-MB-231 breast cancer cells were aligned with nanopatterned substrates comprising long parallel ridges and grooves [23]. Furthermore, the migration speed of C2C12 myoblast cells was higher on culture substrates that topographically restrict sites of cell attachment and align adhesions [22]. Therefore, it can be speculated that the alignment of FAs, which promotes the directional migration and increases the migration speed of the IMR32 cells, is regulated by the alignment of actin filaments. This hypothesis is supported by the result of the MYCN-siRNA transfection experiment, in which the decrease in angle variance did not occur, and accordingly the directionality, persistence distance and migration speed in cells transfected with the MYCN-siRNA were smaller (figure 5*e*,*k*–*m*). This suggests that the alignment of FAs did not occur when the MYCN-siRNA was added, since the alignment of actin filaments did not occur. This idea is supported by the result that the cells exhibited a non-polarized cell shape when the MYCN-siRNA was added (figure 5*c*).

**Figure 6.**
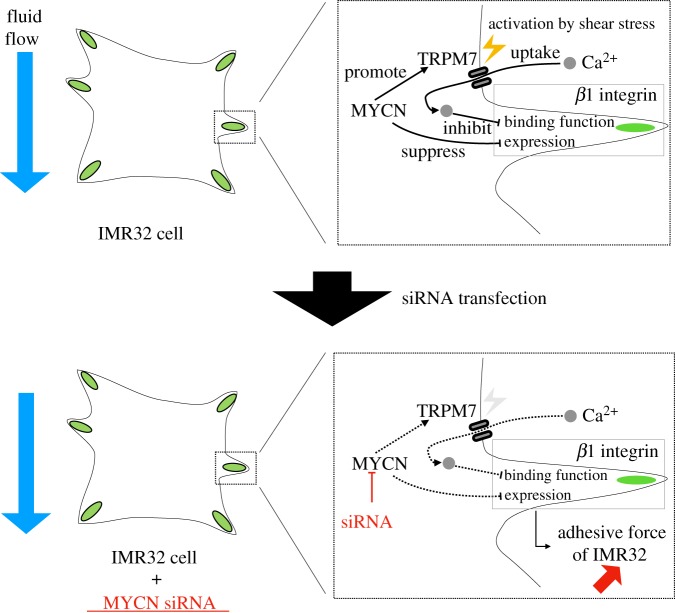
The anticipated relationship between MYCN knockdown and the adhesive forces of IMR32 cells. The upper left panel shows IMR32 cells without MYCN-siRNA transfection. The upper right panel is an enlarged schematic of the boxed region in the left figure. MYCN expression promotes TRPM7 ion channel expression, which is directly activated by shear stress, and suppresses the expression of *β*1 integrin. The TRPM7 ion channel is responsible for uptake of extracellular Ca^2+^ ions. Intracellular Ca^2+^ ions decrease the *α*5*β*1 integrin-mediated adhesion force. The lower panels are a schematic of MYCN knockdown, increasing the adhesive force of IMR32 cells. (Online version in colour.)

Cells transfected with MYCN-siRNA exhibited FAs that were isotropically distributed under high shear stress (figure 5*c*,*d*). We hypothesize that the isotropic distribution of FAs is caused by an increase in cell adhesive force (figure 6). This could be mediated by N-Myc protein, which suppresses the expression of *α*1, *α*2, *α*3 and *β*1 integrin subunits, promoting cell adhesive force [18,24]. MYCN-siRNA weakens this suppressive function of MYCN on integrin subunits, and as a result, cell adhesive force may increase. Another factor that decreases cell adhesive force in IMR32 cells is elevation of intracellular Ca^2+^ concentration. Calcium cation/calmodulin-dependent serine/threonine protein kinase type II directly phosphorylates integrin cytoplasmic domain-associated protein 1*α*, which inactivates *β*1 integrin [51]. Thus, elevation of the intracellular Ca^2+^ concentration decreases cell adhesive force. Shear stress-induced uptake of Ca^2+^ is mediated by transient receptor potential cation channel subfamily M, member 7 (TRPM7), transient receptor potential cation channel subfamily V, member 4 and P2X7 [52,53]. In recent years, it has been reported that the expression of TRPM7 is promoted by N-Myc protein in neuroblastoma cells [54,55]. TRPM7 channels accumulate at the plasma membrane with shear stress values in the range of 0.0–2.0 Pa [56]. This accumulation may cause an increase in the intracellular Ca^2+^ concentration in response to shear stress. By combining these findings, we may propose that the cell adhesive force of IMR32 cells is maintained under high shear stress because Ca^2+^ uptake is decreased, and this is due to the decrease in TRPM7 expression that is induced by MYCN-siRNA transfection. Therefore, it is possible that IMR32 cell FAs in the upstream region could not detach from the substrate under high shear stress, due to the increased cell adhesive force when MYCN-siRNA was added. MYCN-siRNA transfection affects measured parameters (angle variance, breadth, displacement, directionality, persistence distance and migration speed) compared to negative control siRNA only at a 0.4 Pa level of shear stress (figure 5). A possible explanation is that shear stress of a particular threshold is required for the activation of shear-sensitive Ca^2+^ permeable channels [57,58].

We designed and produced the logarithmic flow-rate device. It enabled us to efficiently investigate the effects of shear stress on cells in a two-dimensional culture. However, the cell motility and the forces exerted on cells differ between two- and three-dimensional cultures [6,59]. Therefore, it will be important to develop a system like this for three-dimensional culture in the future.

In our study, we found that high shear stress increases the directional migration and migration speed of IMR32 cells. We confirmed that these phenomena are regulated by morphological changes in FAs, which are promoted by shear stress. Furthermore, we found that expression of MYCN protein was related to the motility of IMR32 cells under high shear stress. These findings suggest that MYCN-amplified neuroblastoma cells migrate towards lymphatic vessels and venules as a result of mechanical stimulation *in vivo*.

## Supplementary Material

The spatial distribution of FAs under shear stress.

## Data accessibility

The authors confirm that the data support the findings of this study. Data are exemplarily shown within the article and its electronic supplementary material. Additional data that support the findings of this study are available from the corresponding author upon request.

## References

[1] Clark AG, Vignjevic DM 2015 Modes of cancer cell invasion and the role of the microenvironment. Curr. Opin. Cell Biol. 36, 13–22. (10.1016/j.ceb.2015.06.004) 26183445

[2] Pereira ER *et al.* 2018 Lymph node metastases can invade local blood vessels, exit the node, and colonise distant organs in mice. Science 359, 1403–1407. (10.1126/science.aal3622) 29567713 PMC6002772

[3] Lawler K, Foran E, O’Sullivan G, Long A, Kenny D 2006 Mobility and invasiveness of metastatic esophageal cancer are potentiated by shear stress in a ROCK- and Ras-dependent manner. Am. J. Physiol. Cell Physiol. 291, C668–C677. (10.1152/ajpcell.00626.2005) 16641163

[4] Shields JD, Fleury ME, Yong C, Tomei AA, Randolph GJ, Swartz MA 2007 Autologous chemotaxis as a mechanism of tumor cell homing to lymphatics via interstitial flow and autocrine CCR7 signaling. Cancer Cell 11, 526–538. (10.1016/j.ccr.2007.04.020) 17560334

[5] Mitchell MJ, King MR 2013 Computational and experimental models of cancer cell response to fluid shear stress. Front. Oncol. 3, 44 (10.3389/fonc.2013.00044) 23467856 PMC3587800

[6] Polacheck WJ, German AE, Mammoto A, Ingber DE, Kamm RD 2014 Mechanotransduction of fluid stresses governs 3D cell migration. Proc. Natl Acad. Sci USA 111, 2447–2452. (10.1073/pnas.1316848111) 24550267 PMC3932905

[7] Munson JM, Shieh AC 2014 Interstitial fluid flow in cancer: implications for disease progression and treatment. Cancer Manage. Res. 6, 317–328. (10.2147/CMAR.S65444) PMC4144982 25170280

[8] Chang SF, Chang CA, Lee DY, Lee PL, Yeh YM, Yeh CR, Cheng CK, Chien S, Chiu JJ 2008 Tumor cell cycle arrest induced by shear stress: roles of integrins and Smad. Proc. Natl Acad. Sci. USA 105, 3927–3932. (10.1073/pnas.0712353105) 18310319 PMC2268796

[9] Lee HJ, Diaz MF, Price KM, Ozuna JA, Zhang S, Sevick-Muraca EM, Hagan JP, Wenzel PL 2017 Fluid shear stress activates YAP1 to promote cancer cell motility. Nat. Commun. 8, 14122 (10.1038/ncomms14122) 28098159 PMC5253685

[10] Avraham-Chakim L, Elad D, Zaretsky U, Kloog Y, Jaffa A, Grisaru D 2013 Fluid-flow induced wall shear stress and epithelial ovarian cancer peritoneal spreading. PLoS ONE 8, e60965 (10.1371/journal.pone.0060965) 23593358 PMC3622607

[11] Xiong N, Li S, Tang K, Bai H, Peng Y, Yang H, Wu C, Liu Y 2017 Involvement of caveolin-1 in low shear stress-induced breast cancer cell motility and adhesion: roles of FAK/Src and ROCK/p-MLC pathways. Biochim. Biophys. Acta (BBA) 1864, 12–22. (10.1016/j.bbamcr.2016.10.013) 27773611

[12] Mitchell MJ, King MR 2013 Fluid shear stress sensitizes cancer cells to receptor-mediated apoptosis via trimeric death receptors. New J. Phys. 15, 015008 (10.1088/1367-2630/15/1/015008) PMC4124740 25110459

[13] DuBois SG *et al.* 1999 Metastatic sites in stage IV and IVS neuroblastoma correlate with age, tumor biology, and survival. J. Pediatr. Hematol. Oncol. 21, 181–189. (10.1097/00043426-199905000-00005) 10363850

[14] Schwankhaus N, Gathmann C, Wicklein D, Riecken K, Schumacher U, Valentiner U 2014 Cell adhesion molecules in metastatic neuroblastoma models. Clin. Exp. Metastasis 31, 483–496. (10.1007/s10585-014-9643-8) 24549749

[15] Huang M, Weiss WA 2013 Neuroblastoma and MYCN. Cold Spring Harb. Perspect. Med. 3, a014415 (doi:0.1101/cshperspect.a014415) 24086065 10.1101/cshperspect.a014415 PMC3784814

[16] Khanna C, Jaboin JJ, Drakos E, Tsokos M, Thiele CJ 2002 Biologically relevant orthotopic neuroblastoma xenograft models: primary adrenal tumor growth and spontaneous distant metastasis. In Vivo (Athens, Greece) 16, 77–85. 12073775

[17] Zaizen Y, Taniguchi S, Noguchi S, Suita S 1993 The effect of N-myc amplification and expression on invasiveness of neuroblastoma cells. J. Pediatr. Surg. 28, 766–769. 8331499 10.1016/0022-3468(93)90321-b

[18] Tanaka N, Fukuzawa M 2008 MYCN downregulates integrin *α*1 to promote invasion of human neuroblastoma cells. Int. J. Oncol. 33, 815–821. 18813796

[19] Wozniak MA, Modzelewska K, Kwong L, Keely PJ 2004 Focal adhesion regulation of cell behavior. Biochim. Biophys. Acta (BBA) 1692, 103–119. (10.1016/j.bbamcr.2004.04.007) 15246682

[20] Kanchanawong P, Shtengel G, Pasapera AM, Ramko EB, Davidson MW, Hess HF, Waterman CM 2010 Nanoscale architecture of integrin-based cell adhesions. Nature 468, 580–584. (10.1038/nature09621) 21107430 PMC3046339

[21] Kim DH, Wirtz D 2013 Focal adhesion size uniquely predicts cell migration. FASEB J. 27, 1351–1361. (10.1096/fj.12-220160) 23254340 PMC3606534

[22] Sheets K, Wunsch S, Ng C, Nain AS 2013 Shape-dependent cell migration and focal adhesion organization on suspended and aligned nanofiber scaffolds. Acta Biomater. 9, 7169–7177. (10.1016/j.actbio.2013.03.042) 23567946

[23] Ray A, Lee O, Win Z, Edwards RM, Alford PW, Kim DH, Provenzano PP 2017 Anisotropic forces from spatially constrained focal adhesions mediate contact guidance directed cell migration. Nat. Commun. 8, 14923 (10.1038/ncomms14923) 28401884 PMC5394287

[24] Judware R, Culp LA 1997 Concomitant down-regulation of expression of integrin subunits by N-myc in human neuroblastoma cells: differential regulation of *α*2, *α*3 and *β*1. Oncogene 14, 1341–1350. (10.1038/sj.onc.1200955) 9178894

[25] Beierle EA, Massoll NA, Hartwich J, Kurenova EV, Golubovskaya VM, Cance WG, McGrady P, London WB 2008 Focal adhesion kinase expression in human neuroblastoma: immunohistochemical and real-time PCR analyses. Clin. Cancer Res. 14, 3299–3305. (10.1158/1078-0432.CCR-07-1511) 18519756

[26] Meyer A, van Golen CM, Kim B, van Golen KL, Feldman EL 2004 Integrin expression regulates neuroblastoma attachment and migration. Neoplasia 6, 332–342. (10.1593/neo.03445) 15256055 PMC1502107

[27] Megison ML, Stewart JE, Nabers HC, Gillory LA, Beierle EA 2013 FAK inhibition decreases cell invasion, migration and metastasis in MYCN amplified neuroblastoma. Clin. Exp. Metastasis 30, 555–568. (10.1007/s10585-012-9560-7) 23208732 PMC3625446

[28] Michael KE, Dumbauld DW, Burns KL, Hanks SK, García AJ 2009 Focal adhesion kinase modulates cell adhesion strengthening via integrin activation. Mol. Biol. Cell 20, 2508–2519. (10.1091/mbc.E08-01-0076) 19297531 PMC2675629

[29] Kim L, Vahey MD, Lee HY, Voldman J 2006 Microfluidic arrays for logarithmically perfused embryonic stem cell culture. Lab Chip 6, 394–406. (10.1039/b511718f) 16511623

[30] Nara K, Kusafuka T, Yoneda A, Oue T, Sangkhathat S, Fukuzawa M 2007 Silencing of MYCN by RNA interference induces growth inhibition, apoptotic activity and cell differentiation in a neuroblastoma cell line with MYCN amplification. Int. J. Oncol. 30, 1189–1196. (10.3892/ijo.30.5.1189) 17390021

[31] Ovcharenko D, Jarvis R, Hunicke-Smith S, Kelnar K, Brown D 2005 High-throughput RNAi screening in vitro: from cell lines to primary cells. RNA 11, 985–993. (10.1261/rna.7288405) 15923380 PMC1370783

[32] Chung BG, Park JW, Hu JS, Huang C, Monuki ES, Jeon NL 2007 A hybrid microfluidic-vacuum device for direct interfacing with conventional cell culture methods. BMC Biotechnol. 7, 60 (10.1186/1472-6750-7-60) 17883868 PMC2071914

[33] Meinhart CD, Wereley ST, Santiago JG 1999 PIV measurements of a microchannel flow. Exp. Fluids 27, 414–419. (10.1007/s003480050366)

[34] Tseng Q, Duchemin-Pelletier E, Deshiere A, Balland M, Guillou H, Filhol O, Théry M 2012 Spatial organization of the extracellular matrix regulates cell–cell junction positioning. Proc. Natl Acad. Sci. USA 109, 1506–1511. (10.1073/pnas.1106377109) 22307605 PMC3277177

[35] Schindelin J *et al.* 2012 Fiji: an open-source platform for biological-image analysis. Nat. Methods 9, 676–682. (10.1038/nmeth.2019) 22743772 PMC3855844

[36] Sternberg SR 1983 Biomedical image processing. Computer 16, 22–34. (10.1109/MC.1983.1654163)

[37] Otsu N 1979 A threshold selection method from gray-level histograms. IEEE Trans. Syst. Man Cybern. 9, 62–66. (10.1109/TSMC.1979.4310076)

[38] Soille P, Vincent LM 1990 Determining watersheds in digital pictures via flooding simulations. Proc. SPIE 1360, 240–251. (10.1117/12.24211)

[39] Fisher NI 1995 Statistical analysis of circular data. Cambrige, UK: Cambridge University Press.

[40] Hothorn T, Bretz F, Westfall P 2008 Simultaneous inference in general parametric models. Biom. J. 50, 346–363. (10.1002/bimj.200810425) 18481363

[41] Skelley AM, Voldman J 2008 An active bubble trap and debubbler for microfluidic systems. Lab Chip 8, 1733–1737. (10.1039/b807037g) 18813398

[42] Booth R, Noh S, Kim H 2014 A multiple-channel, multiple-assay platform for characterization of full-range shear stress effects on vascular endothelial cells. Lab Chip 14, 1880–1890. (10.1039/c3lc51304a) 24718713

[43] Cimetta E, Cannizzaro C, James R, Biechele T, Moon RT, Elvassore N, Vunjak-Novakovic G 2010 Microfluidic device generating stable concentration gradients for long term cell culture: application to Wnt3a regulation of *β*-catenin signaling. Lab Chip 10, 3277–3283. (10.1039/c0lc00033g) 20936235 PMC4106280

[44] Ridley AJ, Schwartz MA, Burridge K, Firtel RA, Ginsberg MH, Borisy G, Parsons JT, Horwitz AR 2003 Cell migration: integrating signals from front to back. Science 302, 1704–1709. (10.1126/science.1092053) 14657486

[45] Deramaudt TB, Dujardin D, Hamadi A, Noulet F, Kolli K, De Mey J, Takeda K, Rondé P 2011 FAK phosphorylation at Tyr-925 regulates cross-talk between focal adhesion turnover and cell protrusion. Mol. Biol. Cell 22, 964–975. (10.1091/mbc.E10-08-0725) 21289086 PMC3069021

[46] Malek AM, Alper SL, Izumo S 1999 Hemodynamic shear stress and its role in atherosclerosis. JAMA 282, 2035–2042. (10.1001/jama.282.21.2035) 10591386

[47] Mitchell MJ, Denais C, Chan MF, Wang Z, Lammerding J, King MR 2015 Lamin A/C deficiency reduces circulating tumor cell resistance to fluid shear stress. Am. J. Physiol. Cell Physiol. 309, C736–C746. (10.1152/ajpcell.00050.2015) 26447202 PMC4725441

[48] Onoue N, Nawata J, Tada T, Zhulanqiqige D, Wang H, Sugimura K, Fukumoto Y, Shirato K, Shimokawa H 2008 Increased static pressure promotes migration of vascular smooth muscle cells: involvement of the Rho-kinase pathway. J. Cardiovasc. Pharmacol. 51, 55–61. (10.1097/FJC.0b013e31815b9d26) 18209569

[49] Tzima E, Del Pozo MA, Shattil SJ, Chien S, Schwartz MA 2001 Activation of integrins in endothelial cells by fluid shear stress mediates Rho-dependent cytoskeletal alignment. EMBO J. 20, 4639–4647. (10.1093/emboj/20.17.4639) 11532928 PMC125600

[50] Soiné JRD, Brand CA, Stricker J, Oakes PW, Gardel ML, Schwarz US 2015 Model-based traction force microscopy reveals differential tension in cellular actin bundles. PLoS Comput. Biol. 11, e1004076 (10.1371/journal.pcbi.1004076) 25748431 PMC4352062

[51] Millon-Frémillon A, Brunner M, Abed N, Collomb E, Ribba A, Block MR, Albigès-Rizo C, Bouvard D 2013 Calcium and calmodulin-dependent serine/threonine protein kinase type II (CaMKII)-mediated intramolecular opening of integrin cytoplasmic domain-associated protein-1 (ICAP-1*α*) negatively regulates *β*1 integrins. J. Biol. Chem. 288, 20 248–20 260. (10.1074/jbc.M113.455956) 23720740 PMC3711292

[52] Wei C, Wang X, Chen M, Ouyang K, Song LS, Cheng H 2009 Calcium flickers steer cell migration. Nature 457, 901–905. (10.1038/nature07577) 19118385 PMC3505761

[53] Hope JM, Greenlee JD, King MR 2018 Mechanosensitive ion channels: TRPV4 and P2X7 in disseminating cancer cells. Cancer J. 24, 84–92. (10.1097/PPO.0000000000000312) 29601335 PMC5880301

[54] Lange I, Koomoa DLT 2014 MycN promotes TRPM7 expression and cell migration in neuroblastoma through a process that involves polyamines. FEBS Open Bio 4, 966–975. (10.1016/j.fob.2014.10.012) PMC4241534 25426416

[55] Zhang Z, Faouzi M, Huang J, Geerts D, Yu H, Fleig A, Penner R 2014 N-Myc-induced up-regulation of TRPM6/TRPM7 channels promotes neuroblastoma cell proliferation. Oncotarget 5, 7625–7634. (10.18632/oncotarget.2283) 25277194 PMC4202149

[56] Oancea E, Wolfe JT, Clapham DE 2006 Functional TRPM7 channels accumulate at the plasma membrane in response to fluid flow. Circ. Res. 98, 245–253. (10.1161/01.RES.0000200179.29375.cc) 16357306

[57] Schwarz G, Callewaert G, Droogmans G, Nilius B 1992 Shear stress-induced calcium transients in endothelial cells from human umbilical cord veins. J. Physiol. 458, 527–538. (10.1113/jphysiol.1992.sp019432) 1338792 PMC1175170

[58] Das T, Maiti TK, Chakraborty S 2008 Traction force microscopy on-chip: shear deformation of fibroblast cells. Lab Chip 8, 1308–1318. (10.1039/B803925A) 18651073

[59] Baker BM, Chen CS 2012 Deconstructing the third dimension—how 3D culture microenvironments alter cellular cues. J. Cell Sci. 124, 3015–3024. (10.1242/jcs.079509) PMC3434846 22797912

