# Supplementary material for: Activation of cell migration via morphological changes in focal adhesions depends on shear stress in MYCN-amplified neuroblastoma cells
Source: J R Soc Interface. 2019 Mar 6;16(152):20180934. doi: 10.1098/rsif.2018.0934 (PMC6451396; doi:10.1098/rsif.2018.0934)
Supplement: The spatial distribution of FAs under shear stress. [file rsif20180934supp1.pdf]

Activation of cell migration via morphological changes in focal adhesions depends on shear stress in MYCN-amplified neuroblastoma cells

Takumi Hiraiwa[1], Takahiro G. Yamada[1], Norihisa Miki Ph.D.[2], Akira Funahashi Ph.D.[1], and Noriko Hiroi Ph.D.[3]

[1]Department of Biosciences and Informatics, Keio University, Kanagawa, Japan
[2]Department of Mechanical Engineering, Keio University, Kanagawa, Japan
[3]Department of Pharmacy, Sanyo-onoda city University, Yamaguchi, Japan

1 Supplementary Figure

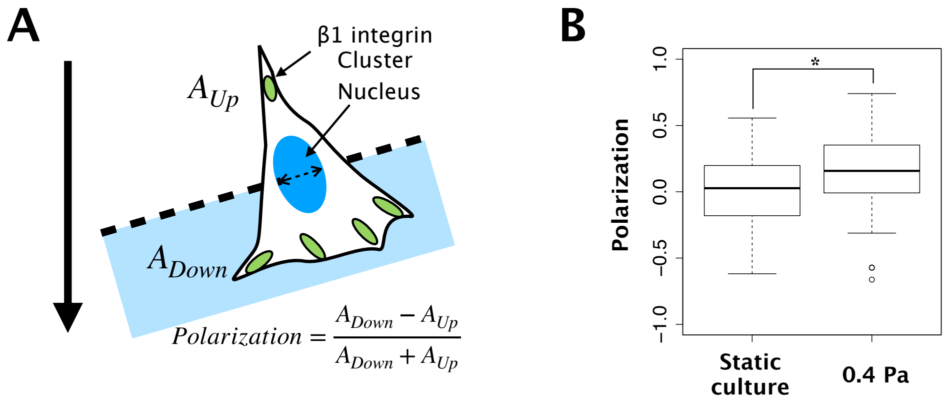

Figure S1: **The spatial distribution of FAs under shear stress.** (A) Quantification of the spatial distribution of $\beta 1$ integrin. The minor axis of the nucleus (black dashed arrow) was detected by ellipse fitting. A_{Up} and A_{Down} is the total area of clusters that are distributed upstream and downstream relative to the extension line (black dashed line) of the minor axis, respectively. Polarization score is calculated using the equation shown at the bottom. To obtain the polarization score, A_{Down} was subtracted from A_{Up} and divided by the total area of the clusters. (B) Polarization score of $\beta 1$ integrin under control and shear stress conditions. We used a Welch's t-test (static culture, n = 35; 0.4 Pa, n = 39 cells). Box, horizontal axis, error bar, and whiskers shows the interquartile range (first quartile to third quartile), the median, the inner fence, and the outliers, respectively. * $p < 0.05$.
